# DNA exonuclease Trex1 regulates radiotherapy-induced tumour immunogenicity

**DOI:** 10.1038/ncomms15618

**Published:** 2017-06-09

**Authors:** Claire Vanpouille-Box, Amandine Alard, Molykutty J. Aryankalayil, Yasmeen Sarfraz, Julie M. Diamond, Robert J. Schneider, Giorgio Inghirami, C. Norman Coleman, Silvia C. Formenti, Sandra Demaria

**Affiliations:** 1 Department of Radiation Oncology, Weill Cornell Medicine, 1300 York Avenue, Box 169, New York, New York 10065, USA; 2 Department of Microbiology, New York University School of Medicine, 450 29th Street, New York, New York 10016, USA; 3 Radiation Oncology Branch, Center for Cancer Research and Radiation Research Program, Division of Cancer Treatment and Diagnosis, National Cancer Institute, NIH, Building #10, Room B3 B406, 9000 Rockville Pike, Bethesda, Maryland 20892, USA; 4 Department of Pathology and Laboratory Medicine, Weill Cornell Medicine, 525 East 68th Street, New York, New York 10065, USA; 5 Present address: Cancer Research Center of Toulouse (CRCT), INSERM UMR 1037-University Toulouse III Paul Sabatier, Tumor Biology Department, Toulouse 31062, France

## Abstract

Radiotherapy is under investigation for its ability to enhance responses to immunotherapy. However, the mechanisms by which radiation induces anti-tumour T cells remain unclear. We show that the DNA exonuclease Trex1 is induced by radiation doses above 12–18 Gy in different cancer cells, and attenuates their immunogenicity by degrading DNA that accumulates in the cytosol upon radiation. Cytosolic DNA stimulates secretion of interferon-β by cancer cells following activation of the DNA sensor cGAS and its downstream effector STING. Repeated irradiation at doses that do not induce Trex1 amplifies interferon-β production, resulting in recruitment and activation of Batf3-dependent dendritic cells. This effect is essential for priming of CD8^+^ T cells that mediate systemic tumour rejection (abscopal effect) in the context of immune checkpoint blockade. Thus, Trex1 is an upstream regulator of radiation-driven anti-tumour immunity. Trex1 induction may guide the selection of radiation dose and fractionation in patients treated with immunotherapy.

## Abstract (web summary)

Trex1 is an exonuclease that degrades cytosolic DNA and has been associated with modulation of interferon responses in autoimmunity and viral infections. Here, the authors show that Trex1 attenuates the immunogenicity of cancer cells treated with high radiation doses by degrading cytosolic DNA and preventing the activation of interferon response.

Treatment with antibodies that target regulatory receptors cytotoxic T-lymphocyte-associated protein 4 (CTLA4) and programmed death-1 (PD-1) in T cells to improve their activation, and effector function induces durable responses in a variable percentage of patients with metastatic disease across different malignancies^1^. However, the majority of patients does not respond to the blockade of these immune checkpoints, often because their tumours are less immunogenic and do not elicit a sufficient immune reaction^1^. Thus, to enhance responses it is necessary to identify treatments that synergize with immune checkpoints inhibitors (ICI) by stimulating anti-tumour T cell responses to poorly immunogenic tumours.

Radiotherapy is under investigation in the clinic for its ability to induce anti-tumour T cells, and enhance responses to immune checkpoint inhibitors and other immunotherapies^2^,^3^,^4^,^5^,^6^. A variety of radiation doses, fractionation and delivery schedules have been used to induce anti-tumour T cells in preclinical studies^7^,^8^,^9^. However, in the absence of a mechanistic understanding of the relationship between the dose and fractionation of radiation and its immunogenicity, most clinical trials testing the ability of radiation to enhance responses to immunotherapy are guided by standard-of-care or empirical choices that may not be optimal^10^.

Here we report the results of our studies that identify the DNA exonuclease Trex1 as an upstream regulator of radiation-induced anti-tumour immunity, and show that Trex1 expression is dependent on the radiation dose. When radiation is delivered at high dose in a single fraction, with a threshold that varies between 12 and 18 Gy in different cancer cells, Trex1 is induced at levels sufficient to degrade the DNA that accumulates in the cytosol of irradiated cancer cells precluding activation of the type-I interferon (IFN-I) pathway mediated via cyclic GMP-AMP (cGAMP) synthase (cGAS) and its downstream adaptor stimulator of interferon genes (STING)^11^. In contrast, radiation given in repeated doses below the dose threshold for Trex1 induction optimally stimulates the cancer cells to produce IFNβ, required to recruit to the tumour and activate Batf3-dependent dendritic cells (DCs). The latter are essential for priming of tumour-specific CD8^+^ T cells that, in the presence of immune checkpoint inhibitors, mediate complete durable regression of the irradiated and non-irradiated tumour (abscopal effect). These data have important implications for the choice of radiation dose and fractionation in the clinic to convert unresponsive patients into responders to immunotherapy.

## Results

### Abscopal responses are not induced by high dose radiation

**Figure 1:**
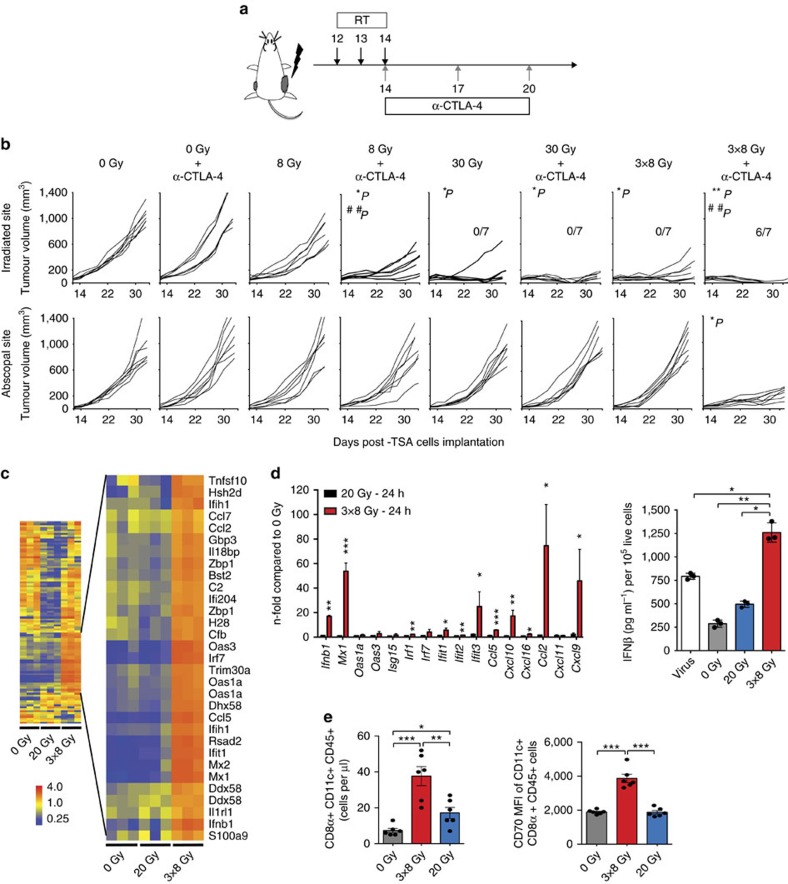
Radiation-induced activation of type-I interferon pathway correlates with radiation's ability to induce abscopal responses in combination with anti-CTLA4. (**a**,**b**) In mice with bilateral TSA tumours one tumour was irradiated (RT) and mice received anti-CTLA4 antibody as indicated (**a**). Growth of irradiated and abscopal tumours in mice treated with 0 Gy, 0 Gy+anti-CTLA4, 8GyX1, 8GyX1+anti-CTLA4, 30GyX1, 30GyX1+anti-CTLA4, 8GyX3 and 8GyX3+anti-CTLA4. Ratios indicate the number of mice free from the irradiated tumour. (Duplicate; asterisks indicate *P* values for the comparison of irradiated tumours in each group versus 0 Gy controls, *P<0.05; **P<0.005; hashs indicate *P* values for the comparison of tumours treated or not with anti-CTLA4 within each radiation level, ^##^*P*<0.005; two-way ANOVA; *n*=7). (**b**). (**c**) Heat map of gene expression in TSA tumours 24 h after radiation *in vivo* (*n*=3). (**d**) qRT-PCR (*n*=4) and IFNβ secretion (*n*=3) 24 h after *in vitro* irradiation of TSA cells (Triplicate; *P<0.05; **P<0.005; ***P<0.0005: *t*-test). (**e**) Number of CD11c^+^CD8α^+^ DCs infiltrating TSA tumours 5 days after irradiation and CD70 mean fluorescence intensity (MFI) gated on CD11c^+^CD8α^+^ cells (*n*=5). (Duplicate; *P<0.05; **P<0.005; ***P<0.0005: *t*-test). All data are mean±s.e.m.

To identify the mechanisms by which tumour-directed radiation synergizes with anti-CTLA4 antibody (anti-CTLA4) to induce anti-tumour T cells against poorly immunogenic tumours, we used TSA, a mouse mammary carcinoma refractory to immune checkpoint inhibitors, as a model. We have previously identified an effective regimen, 8 Gy given in three consecutive days (8GyX3), and an ineffective one, 20 Gy single dose, to induce T cell-mediated rejection of irradiated and synchronous non-irradiated TSA tumours with anti-CTLA4 (ref. 12). To determine if a single 8 Gy dose or a higher 30 Gy dose could synergize with anti-CTLA4, mice bearing bilateral TSA tumours received radiation to one tumour and then they were followed for responses in both irradiated and non-irradiated (abscopal) tumours (Fig. 1a). Abscopal responses were only seen in mice treated with 8GyX3 plus anti-CTLA4 (Fig. 1b). In the absence of anti-CTLA4 8GyX3 and 30 Gy were similarly effective at controlling the growth of the irradiated tumour, but complete durable regression of the irradiated tumour was achieved by addition of anti-CTLA4 only in mice treated with 8GyX3. Interestingly, anti-CTLA4 treatment also led to a significant improvement in control of the irradiated tumour in mice treated with 8GyX1. Notably, depletion of CD8^+^ T cells abrogated abscopal responses and complete tumour regression in mice treated with anti-CTLA4 and 8GyX3 (Supplementary Fig. 1a).

### Radiation dose-dependent IFNβ activation in cancer cells

Having confirmed that 20 Gy and 30 Gy single dose were similarly ineffective, and that abscopal effects with anti-CTLA4 could be observed only after repeated 8 Gy doses, we then studied the differences in tumour response to 20 Gy and 8GyX3. First, gene transcripts induced by radiation *in vivo* were analysed in tumours shortly after completion of 8GyX3 and 20 Gy, revealing the differential expression of IFN-I stimulated genes (ISGs), upregulated only by 8GyX3 (Fig. 1c). *In vitro* irradiation of TSA carcinoma cells in the absence of the tumour stroma showed that the upregulation of ISGs was a cancer cell-intrinsic response, and that only virus infection and 8GyX3 but not 20 Gy could induce the release of IFNβ cytokine by TSA cells (Fig. 1d). Similar results were obtained with the breast 4T1 and colorectal MCA38 mouse carcinomas, and with the human breast cancer cells MDA-MB-231 (Supplementary Fig. 1b–e). Although the latter did not show significant increase of *Ifnb1* gene expression, increased secretion of IFNβ was observed in response to 8GyX3. Analysis of tumour-infiltrating DCs (TIDCs) revealed a marked increase in CD8α^+^ TIDCs within TSA tumours after 8GyX3 but not 20 Gy radiation, consistent with the requirement for IFNβ for recruitment to the tumour of CD8α^+^ DCs, essential for optimal priming of anti-tumour CD8^+^ T cells^13^. TIDCs in 8GyX3-treated tumours expressed higher levels of CD70, a key ligand for CD27 during priming of CD8^+^ T cells^14^ (Fig. 1e). Rejection of the irradiated and abscopal TSA tumours in mice treated with 8GyX3+anti-CTLA4 was abrogated in Batf3^−/−^ and interferon-α/β-receptor-1 (IFNAR1)^−/−^ mice, demonstrating the requirement for CD8α^+^ TIDCs responsive to IFNβ (Supplementary Fig. 2a,b). Similarly, rejection of the irradiated and abscopal MCA38 tumours in mice treated with 8GyX3+anti-CTLA4 was abrogated in IFNAR1^−/−^ mice (Supplementary Fig. 2c).

**Figure 2:**
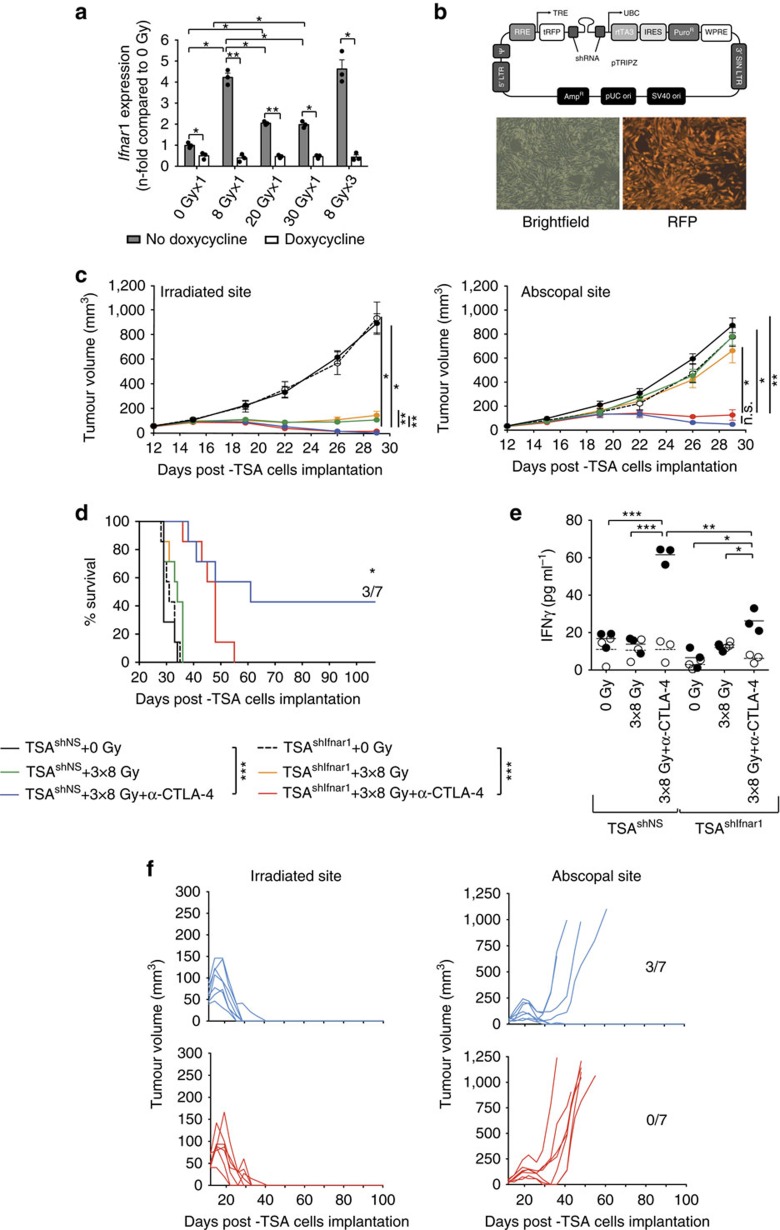
IFNAR1 expression by TSA cancer cells is required for optimal therapeutic response of mice treated with 8GyX3 and anti-CTLA4. (**a**) Upregulation of *Ifnar1* expression measured by qRT-PCR 24 h after radiation *in vitro* in tetracycline-treated TSA^shNS^ cells (black bars) is abrogated in TSA^shIfnar1^ cells (white bars) (Duplicate; *P<0.05; **P<0.005; ***P<0.0005: *t*-test; *n*=3). (**b**) pTRIPZ lentiviral vector with tetracycline-inducible shRNA and TurboRFP fluorescent protein (tRFP) expression, and microscopic image of tetracycline-treated TSA^shNS^ cells (magnification= × 20). (**c**–**f**) Mice bearing irradiated TSA^shIfnar1^ or TSA^shNS^ tumours and TSA^shNS^ tumours in the abscopal site were treated with tetracycline and 0Gy (TSA^shNS^=black; TSA^shIfnar1^=dashed line), 8GyX3 (TSA^shNS^=green; TSA^shIfnar1^=yellow), 8GyX3+anti-CTLA4 (TSA^shNS^=blue; TSA^shIfnar1^=red). (**c**) Growth of irradiated and abscopal tumours. Duplicate; *P<0.05; **P<0.005: comparison of irradiated tumour outgrowth; two-way ANOVA; *n*=7. (**d**) Survival. Numbers indicates fraction of tumour-free mice. Log-rank (Mantel–Cox) test for the survival experiment; *n*=7 (**e**) IFNγ production by tumour-draining lymph node (TDLN) cells in response to the CD8 T cell epitopes AH1A5 (full circles) or control peptide MCMV (open circles). Each symbol represents one animal. Horizontal lines indicate the mean of antigen-specific (solid lines) or control (dashed lines) IFNγ concentration. Duplicate; *P<0.05; **P<0.005; ***P<0.0005: *t*-test; *n*=3 (**f**) Individual tumour growth from (**d**) showing recurrence of all abscopal tumours in mice bearing irradiated TSA^shIfnar1^ tumours (red) while 3/7 mice with irradiated TSA^shNS^ tumours (blue) remained tumour-free. All data are mean±s.e.m.

Induction of IFNAR1 was significantly more robust in TSA cells treated with 8GyX1 and 8GyX3 than 20GyX1 or 30GyX1 radiation (Fig. 2a). To determine if responsiveness to IFNβ by TSA cells was required for the therapeutic effect of 8GyX3+anti-CTLA4, IFNAR1 expression was abrogated in TSA cells expressing an inducible shRNA targeting *Ifnar1* (TSA^shIfnar1^) by feeding mice with doxycycline before tumour irradiation (Fig. 2a,b). Regression of the irradiated and abscopal tumours was similar in mice bearing TSA^shIfnar1^ and control TSA^shNS^ cells at the irradiated site, indicating that responsiveness of cancer cells to IFNβ was not required for 8GyX3+anti-CTLA4-induced tumour inhibition (Fig. 2c). However, mice with IFNβ-unresponsive TSA cells at the irradiated site mounted weaker tumour-specific CD8^+^ T cell responses and did not achieve long-term survival due to impaired ability to control the abscopal tumour, which either did not regress completely or recurred, while 43% of the mice with IFNAR1^+^ TSA cells at the irradiated site remained tumour-free for over 100 days (Fig. 2d-f). Thus, IFNβ responsiveness is required in both, the tumour and the host, for optimal induction of anti-tumour immunity by radiation and anti-CTLA4.

### Trex1 regulates cytoplasmic DNA levels in irradiated cells

**Figure 3:**
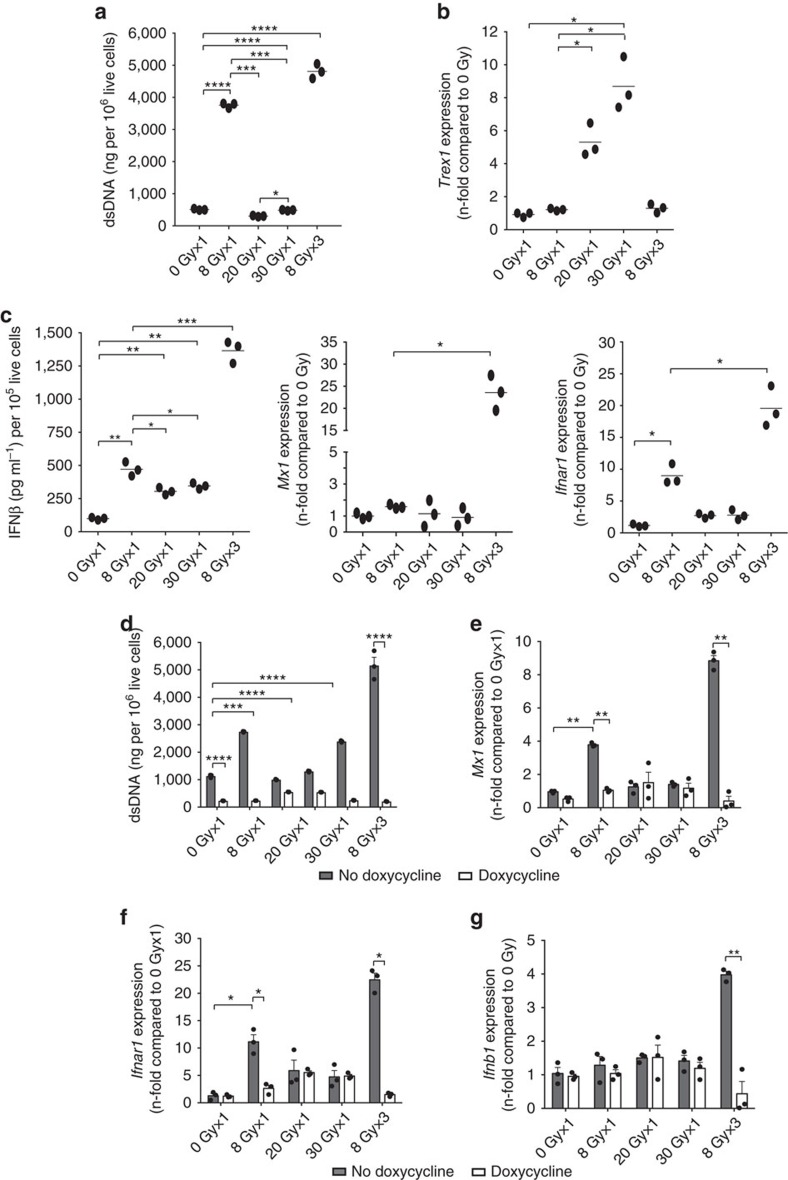
Trex1 is induced by high dose radiation and degrades IFN-inducing cytoplasmic DNA. (**a**–**c**) Cytoplasmic dsDNA accumulation (**a**), *Trex1* gene expression (**b**), IFNβ secretion and *Mx1* and *Ifnar1* gene expression (**c**) in TSA cells treated with various radiation doses (*n*=3). (**d**–**g**) TSA^KI *Trex1*^ cells were cultured without (grey bars) or with doxycycline (white bars) to induce *Trex1* expression (Supplementary Fig. 3b) before measuring cytoplasmic dsDNA accumulation (**d**), *Mx1* (**e**), *Ifnar1* (**f**) and *Ifnb1* (**g**) gene expression induced by radiation (*n*=3). (Duplicate; *P<0.05; **P<0.005; ***P<0.0005; ****P<0.0001: *t*-test). All data are mean±s.e.m.

**Figure 4:**
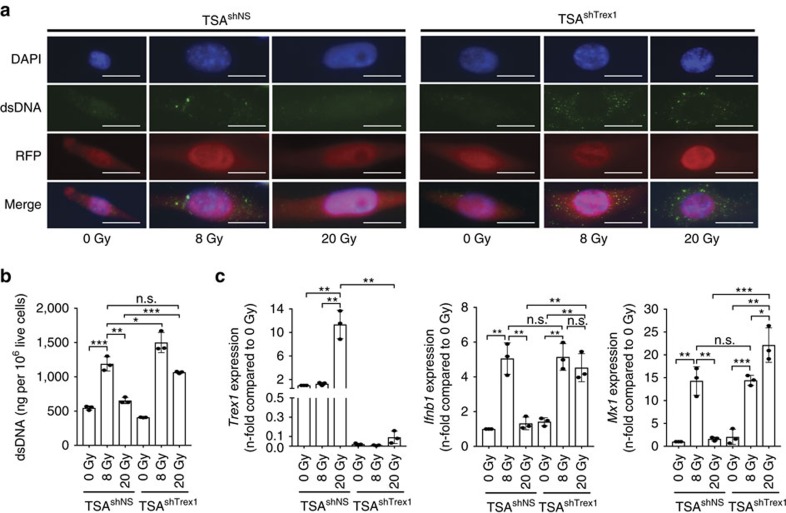
Trex1 knockdown restores cytosolic dsDNA accumulation and induction of ISGs in TSA cells treated with 20 Gy. (**a–c**) TSA^shNS^ and TSA^shTrex1^ cells were treated with doxycycline to induce *Trex1* knockdown and tRFP expression, then exposed to a radiation dose of 8 Gy or 20 Gy. (**a**) After 24 h cells were fixed, permeabilized and stained with an antibody specific for dsDNA, followed by detection with Alexa fluor 488-conjugated secondary antibody. Representative micrographs show DAPI-stained nuclei (blue), cytoplasmic dsDNA (green), cytoplasmic tRFP (red), and the three channels combined. Magnification: × 400; duplicate; *n*=3 per group. White bars, 10 μm. (**b**,**c**) After 24 h cells were harvested for measurement of cytoplasmic dsDNA (**b**) or *Trex1*, *Ifnb1* and *Mx1* expression by qRT-PCR (**c**). Duplicate; *P<0.05; **P<0.005; ***P<0.0005: *t*-test; *n*=3. All data are mean±s.e.m.

IFNβ production by epithelial cells in the absence of viral infection can be triggered by DNA damage, which leads to accumulation of self-DNA into the cytoplasm^15^,^16^. Surprisingly, significantly higher levels of double-stranded DNA (dsDNA) were present in the cytoplasmic fraction of TSA cells treated with one or three 8 Gy radiation doses than with 20 or 30 Gy single doses of radiation (Fig. 3a). The exonuclease Trex1 plays an essential role in clearance of DNA from the cytoplasm of both haematopoietic and non-haematopoietic cells^16^,^17^,^18^, and *Trex1* gene expression can be upregulated by IFN-stimulatory DNA^17^. To determine if Trex1 could control the abundance of cytoplasmic DNA in TSA cells treated with different radiation doses, its expression was analysed. A significant increase of *Trex1* was detected in cells treated with a single dose of 20 and 30 Gy but not 8 Gy, even when given in three consecutive fractions, for a total dose of 24 Gy over 3 days (Fig. 3b). In contrast, release of IFNβ and expression of ISGs *Mx1*, *Ifnar1* and *Cxcl10* were markedly increased only by the 8GyX3 regimen of radiation (Fig. 3c and Supplementary Fig. 3a). Interestingly, while a single dose of 8 Gy induced expression of IFNAR1 and modest levels of IFNβ, repeated irradiation of TSA cells with 8 Gy amplified ISGs induction and IFNβ secretion, explaining the absence of abscopal responses seen with a single dose of 8 Gy+anti-CTLA4 (Fig. 1b). Doxycycline-induced expression of *Trex1* in TSA cells transduced with an inducible *Trex1* cDNA (TSA^KI *Trex1*^) (Supplementary Fig. 3b) abrogated the cytoplasmic accumulation of dsDNA induced by 8GyX3, and the expression of the ISGs *Mx1*, *Ifnar1* and *Cxcl10*, as well as IFNβ release (Fig. 3d–g and Supplementary Fig. 3b). Conversely, doxycycline-inducible shRNA-mediated knockdown of *Trex1* restored the accumulation of dsDNA in the cytoplasm of TSA cells treated with 20 Gy radiation (Fig. 4a,b). Upregulation of *Ifnb1* and *Mx1* induced by 20 Gy in TSA cells with impaired expression of *Trex1* (TSA^shTrex1^) was comparable to the response induced by 8 Gy in TSA cells expressing a non-silencing construct (TSA^shNS^) (Fig. 4c), confirming the key role of Trex1 in downregulating the radiation-induced activation of the IFN type-I pathway.

### cGAS and STING are required for IFN-I induction by radiation

**Figure 5:**
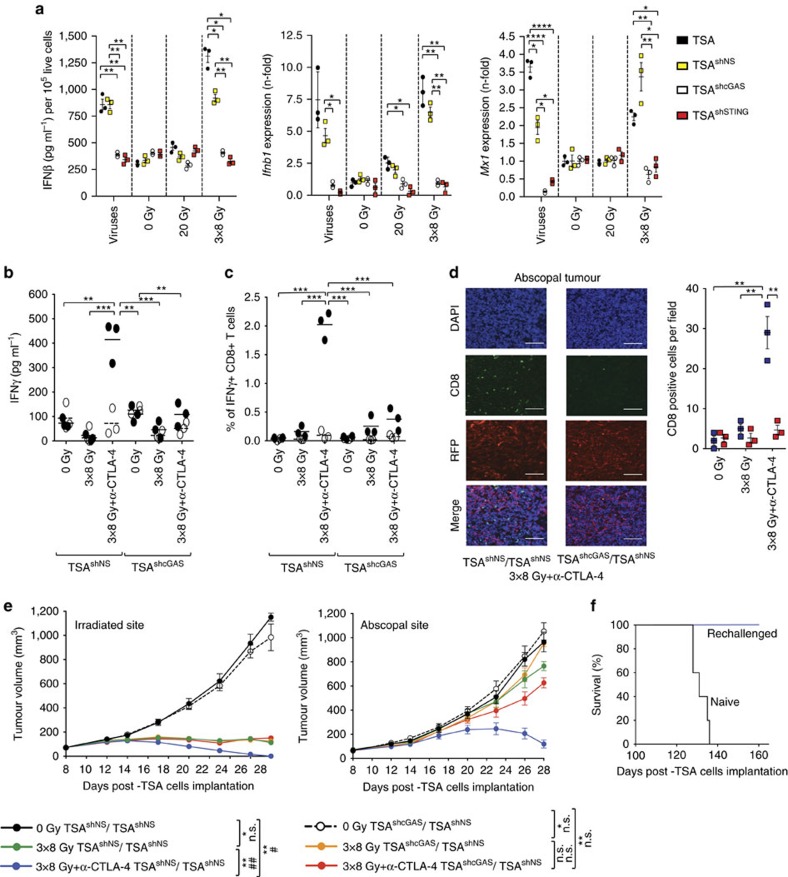
Cancer cell-intrinsic type-I IFN activation is mediated by cGAS-STING pathway and is required for radiation-induced abscopal responses. (**a**) Doxycycline-inducible shRNA-mediated knockdown of cGAS (TSA^shcGAS^) or STING (TSA^shSTING^) in TSA cells completely abrogated IFNβ release and *Ifnb1* and *Mx1* gene expression induced by viral infection and 8GyX3 radiation *in vitro*. (**b**–**f**) Mice with TSA^shcGAS^, or non-silencing shRNA (TSA^shNS^) in the irradiated tumour and TSA^shNS^ in the abscopal tumour were treated with doxycycline, 8GyX3 and anti-CTLA4. IFNγ production by TDLN cells (**b**) and percentage of IFNγ^+^ CD8^+^ T cells in spleen (**c**) in response to CD8 epitope AH1A5 (full circles) or control peptide MCMV (open circles). Each symbol represents one animal. Horizontal lines indicate the mean of antigen-specific (solid lines) or control (dashed lines). (**d**) Representative fields (× 200) showing CD8^+^ cells (green), DAPI+ nuclei (blue), and RFP+ TSA cells (red), and mean number±s.d. of CD8^+^ cells per field in abscopal tumours harvested at day 22 from 8GyX3+anti-CTLA4-treated mice with irradiated TSA^shcGAS^ (red squares) or TSA^shNS^ (blue squares) tumours. White bars, 25 um. (**e**) Growth of irradiated and abscopal tumour in mice with TSA^shNS^ cells treated with 0Gy (black), 8GyX3 (green), 8GyX3+anti-CTLA4 (blue), and mice with TSA^shcGAS^ cells treated with 0Gy (dashed line), 8GyX3 (yellow), 8GyX3+anti-CTLA4 (red). (**f**) Survival of mice from 8GyX3+anti-CTLA4 that rejected the irradiated and abscopal tumour (*n*=4) and were rechallenged at day 100 with a tumorigenic inoculum of TSA cells, together with a group of naïve mice (*n*=5). (**a**-**d**) Duplicate; *P<0.05; **P<0.005; ***P<0.0005: *t*-test; *n*=3. (**e**) Duplicate; *P<0.05; **P<0.005: comparison of irradiated tumour outgrowth; two-way ANOVA; *n*=7; ^##^*P*<0.005: comparison of abscopal tumour outgrowth; two-way ANOVA; *n*=7. All data are mean±s.e.m.

The cyclic GMP-AMP (cGAMP) synthase (cGAS), which activates the downstream adaptor STING via synthesis of 2′-3′-cGAMP leading to induction of ISGs, is the sensor for cytoplasmic double-stranded but not single-stranded DNA^19^,^20^. Activation of cGAS-STING pathway in TIDCs by tumour cell-derived DNA was shown to be involved in the development of natural immune responses to immunogenic tumours, a process amplified by tumour irradiation^21^,^22^. To determine if cGAS-STING pathway plays a role in the cancer cell-intrinsic IFNβ production induced by 8GyX3 radiation doxycycline-inducible shRNA constructs targeting cGAS and STING were introduced in TSA cells (Supplementary Fig. 4a). The knockdown of cGAS or STING completely abrogated IFNβ release and *Ifnβ1* and *Mx1* gene expression induced by viral infection or 8GyX3 treatment of TSA cells *in vitro* (Fig. 5a). Similar results were obtained with 4T1, MCA38 and MDA-MB-231 carcinoma cells (Supplementary Fig. 4b–d). *In vivo*, knockdown of cGAS in TSA cells before the treatment with 8GyX3+anti-CTLA4 abrogated the radiation-induced recruitment and activation of CD8α^+^ TIDCs (Supplementary Fig. 5a,b), the priming of tumour-specific CD8^+^ T cells in tumour-draining lymph nodes and spleen (Fig. 5b,c), and the infiltration of abscopal tumour by CD8^+^ T cells (Fig. 5d). While the response to radiation was not affected by knockdown of cGAS in TSA cells, the improved control achieved in the presence of anti-CTLA4 with complete and durable tumour rejection was abrogated. Likewise, abscopal responses were dependent on the expression of cGAS by TSA cells in the irradiated tumour (Fig. 5e). Mice that rejected the irradiated and abscopal tumours were resistant to a challenge with viable TSA cells, demonstrating long-term immunological memory (Fig. 5f). Similar results were obtained when TSA^shSTING^ tumours were irradiated (Supplementary Fig. 5c–e). Altogether, these results indicate that cancer cell-intrinsic activation of type-I IFN pathway is required for optimal *in situ* vaccination by radiation and anti-CTLA4, and is mediated via cGAS-STING.

### Trex1 regulates the abscopal effects of radiation with ICI

**Figure 6:**
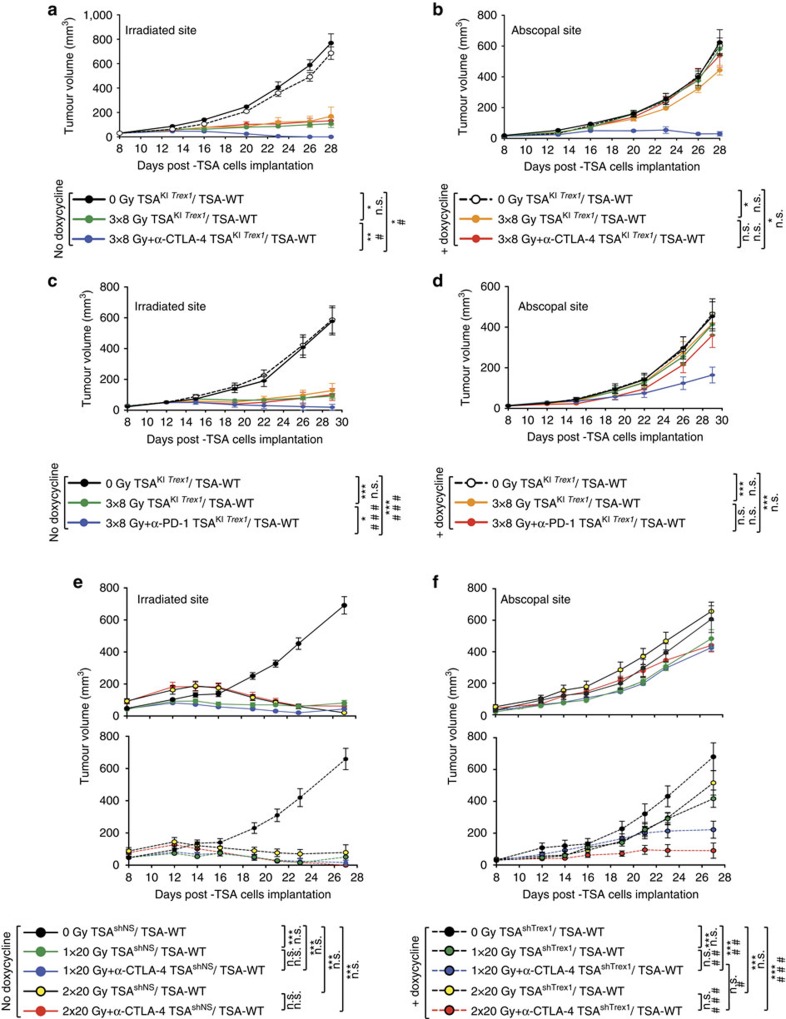
Trex1 regulates radiation-induced abscopal responses in combination with immune checkpoint inhibitors. Half of the mice with doxycycline-inducible *Trex1* in TSA cells (TSA^KI *Trex1*^) in the irradiated tumour and parental TSA (TSA-WT) in the abscopal tumour were given doxycycline and all mice were then treated with 8GyX3 and anti-CTLA4 (**a**,**b**) or anti-PD-1 (**c**,**d**). Growth of irradiated (**a**) and abscopal (**b**) tumour in mice treated with 0 Gy (black), 0 Gy+doxycycline (dashed line), 3 × 8 Gy (green), 3 × 8 Gy+doxycycline (yellow); 3 × 8 Gy+anti-CTLA4 (blue) and 3 × 8 Gy+anti-CTLA4+doxycycline (red). Growth of irradiated (**c**) and abscopal (**d**) tumour in mice treated with 0 Gy (black), 0 Gy+doxycycline (dashed line), 3 × 8 Gy (green), 3 × 8 Gy+doxycycline (yellow); 3 × 8 Gy+anti-PD-1 (blue) and 3 × 8 Gy+anti-PD-1+doxycycline (red). Mice with TSA^shTrex1^ (broken lines) or non-silencing shRNA (TSA^shNS^) (solid lines) in the irradiated tumour and TSA^shNS^ in the abscopal tumour were treated with doxycycline to induce Trex1 knockdown (**e**,**f**). Growth of irradiated (**e**) and abscopal (**f**) tumour in mice treated with 0 Gy (black), 1 × 20 Gy (green), 1 × 20 Gy+anti-CTLA4 (blue), 2 × 20 Gy (yellow), 2 × 20 Gy+anti-CTLA4 (red). (Duplicate; *P<0.05; **P<0.005: comparison of irradiated tumour growth; two-way ANOVA; *n*=7; ^##^*P*<0.005: comparison of abscopal tumour growth; two-way ANOVA; *n*=7). All data are mean±s.e.m.

To further determine if Trex1 is an upstream regulator of the therapeutic synergy of radiation with anti-CTLA4, mice were injected with TSA^KI *Trex1*^ cells at the site to be irradiated and with parental TSA cells in the contralateral flank, and treated or not with doxycycline to induce *Trex1* expression before irradiation with 8GyX3. Doxycycline-mediated induction of *Trex1* completely abrogated the development of tumour-specific CD8 T cell responses in draining lymph nodes (Supplementary Fig. 6), and the complete regression of irradiated tumours as well as abscopal responses were lost (Fig. 6a,b). Similarly, induction of *Trex1* completely abrogated the regression of irradiated tumours as well as abscopal responses in mice treated with 8GyX3 and anti-PD-1 (Fig. 6c,d). Moreover, *Trex1* knockdown in TSA cells in the irradiated tumour restored the ability of radiation used at 20 Gy with anti-CTLA4 to induce abscopal responses (Fig. 6e,f). Abscopal responses were significantly better in *Trex1* knockdown cells with 20GyX2, supporting the requirement for repeated treatment to amplify the response.

### Radiation dose dependency of Trex1 induction in cancer cells

**Figure 7:**
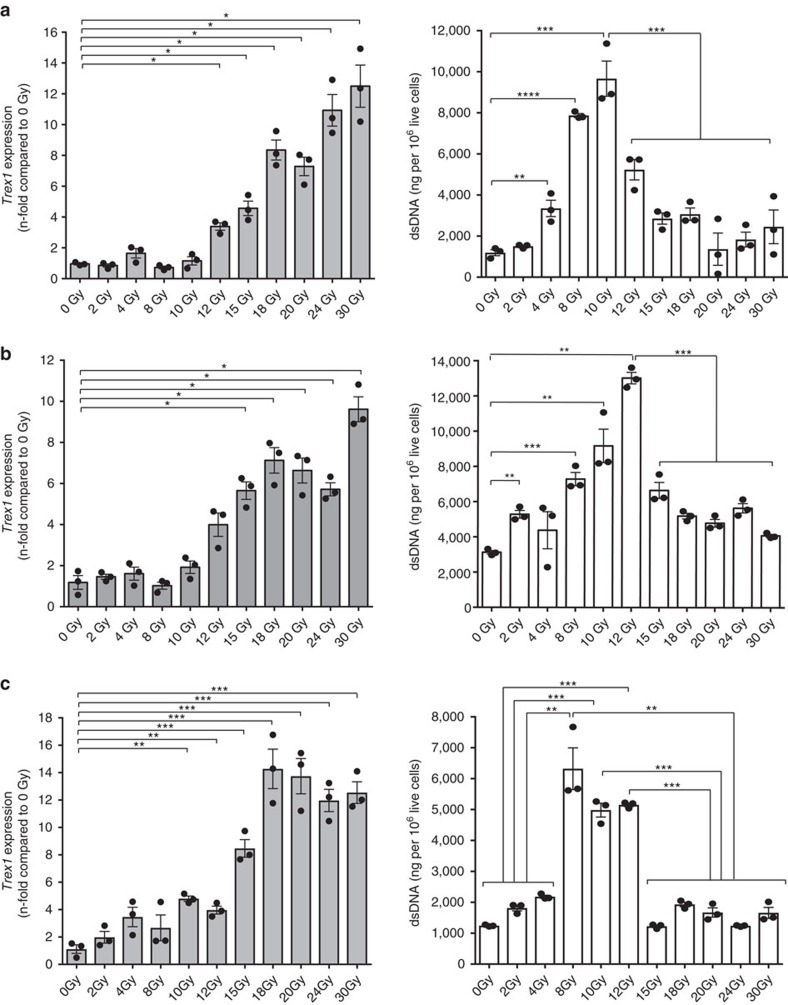
Threshold for Trex1 induction by radiation in mouse cell lines. *Trex1* gene expressions and cytoplasmic dsDNA accumulations in TSA (**a**), MCA38 (**b**) and 4T1 (**c**) carcinoma cells measured 24 h after *in vitro* treatment. Duplicate; *P<0.05; **P<0.005; ***P<0.0005: *t*-test; *n*=3. All data are mean±s.e.m.

**Figure 8:**
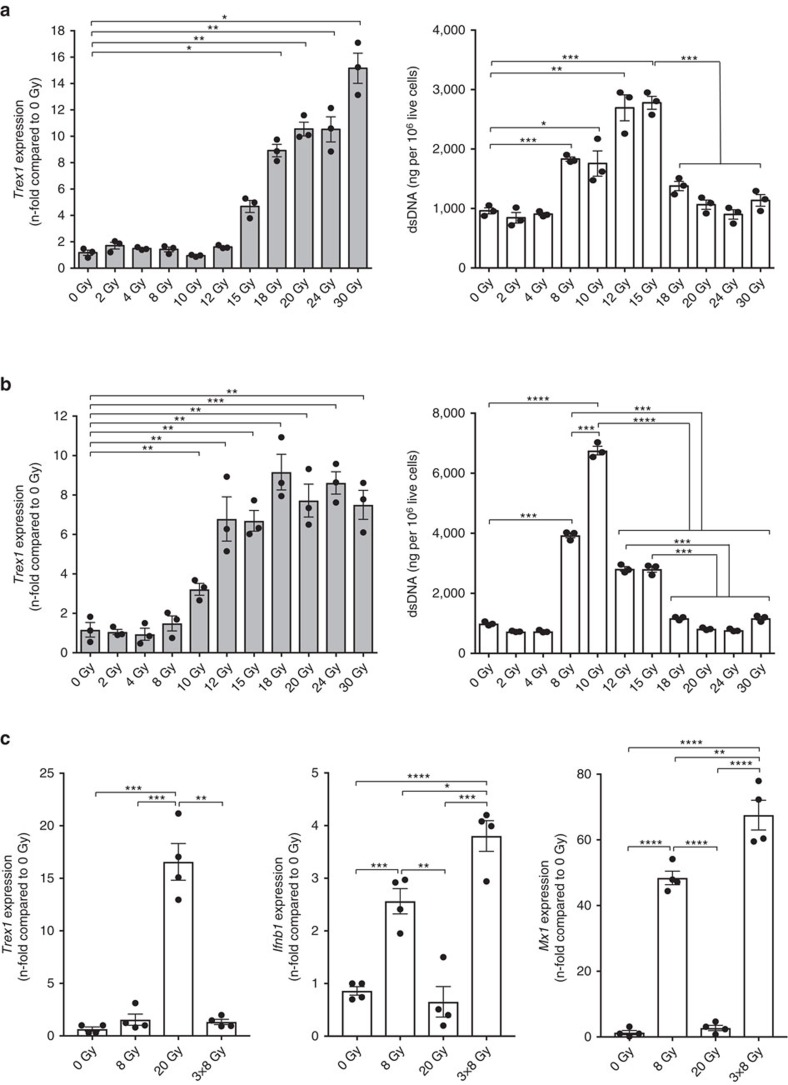
Threshold for Trex1 induction by radiation in human cell lines and induction of Trex1 and IFN-I pathway in a patient-derived lung adenocarcinoma xenograft. (**a**,**b**) *Trex1* expression and cytoplasmic dsDNA accumulations in MDA-MB-231 (**a**) and 4175TR (**b**) breast carcinoma cells 24 hrs after *in vitro* treatment. Duplicate; *P<0.05; **P<0.005; ***P<0.0005: *t*-test; *n*=3. All data are mean±s.e.m. (**c**) *Trex1*, *Ifnb1* and *Mx1* expression measured by qRT-PCR in a patient-derived tumour xenograft (PDTX) 24 h after mice treatment with tumour-directed radiation. Duplicate; *P<0.05; **P<0.005; ***P<0.0005: *t*-test; *n*=4. All data are mean±s.e.m.

To determine if there is a threshold for the induction of Trex1 expression by radiation TSA carcinoma cells were treated with increasing doses of radiation and analysed for cytosolic DNA levels and Trex1 expression. A slight increase in cytosolic dsDNA was measurable in TSA cells treated with 4 Gy and reached a plateau between 8 and 10 Gy radiation (Fig. 7a). *Trex1* levels were significantly increased compared to baseline in cells treated with 12 Gy, and further increased at higher doses, resulting in a significant decrease in cytosolic dsDNA. Similar results were obtained with MCA38 and 4T1 cells, with some differences in the dose threshold at which *Trex1* upregulation was sufficient to markedly decrease cytosolic dsDNA, which was 15 Gy in both MCA38 and 4T1 cells (Fig. 7b,c). In human breast carcinoma cells, MDA-MB-231 *Trex1* expression began to increase only at 15 Gy and reached levels sufficient to markedly decrease cytosolic dsDNA at 18 Gy (Fig. 8a), while in human breast carcinoma cells 4175TR a slight increase in *Trex1* expression was already detectable at 10 Gy and reached levels sufficient to markedly decrease cytosolic dsDNA at 12 Gy (Fig. 8b). Thus, in all cancer cells tested high radiation doses induce the upregulation of *Trex1*, but the threshold dose at which it is sufficient to clear the cytosolic dsDNA varies in different cells between 12 and 18 Gy.

To further confirm the translational relevance of our findings, a patient-derived TP53/KRAS-mutated lung adenocarcinoma xenograft was tested for the ability to upregulate IFN-I pathway and *Trex1* gene expression in response to hypofractionated versus high single dose radiation. Similar to the results obtained with the mouse carcinomas, marked upregulation of human *Ifnb1* and *Mx1* was seen after irradiation with 8 Gy X 1, which was further increased with 8 Gy X 3, while 20 Gy upregulated *Trex1* (Fig. 8c).

## Discussion

We conclude that the cancer cell-intrinsic activation of type-I IFN pathway and production of IFNβ by the cancer cells is an essential mechanism of anti-tumour T cell generation by radiation, which is required to elicit anti-tumour effectors able to mediate abscopal responses. Intratumoral accumulation of Batf3-dependent DCs is required for development of anti-tumour CD8^+^ T cells^13^ and for response to immune checkpoint inhibitors^23^. Importantly, we have previously shown that the number of DCs within 4T1 tumours determined the magnitude of the anti-tumour CD8^+^ T cell response elicited by radiotherapy and anti-CTLA4 (ref. 24). Thus, radiation-induced accumulation of DCs in tumours resistant to immune checkpoint inhibitors underlies radiotherapy's ability to restore tumour responses to these treatments^25^. In addition, upregulated expression of IFN-inducible chemokines *cxcl9*, *cxcl10*, *cxcl11* and *cxcl16* (ref. 26) was also seen in mouse and human tumours following radiation doses unable to induce Trex1 (Fig. 1d and Supplementary Figs 1e and 3a,b). These chemokines likely contribute to the recruitment of effector T cells to the irradiated tumour. In fact, we have previously shown a role for radiation-induced CXCL16 in recruitment of CD8^+^ T cells to 4T1 tumours^27^.

Several cell types in the tumour microenvironment are able to produce IFNβ when properly stimulated, including phagocytes and endothelial cells^21^,^28^. In fact, when MCA38 tumours were treated with 20 Gy, a dose of radiation that is unable to stimulate cancer cell-intrinsic IFNβ production, intratumoral administration of the STING agonist cGAMP was required to improve tumour response^22^. Intra tumorally administered cGAMP was shown to induce IFNβ production mainly by endothelial cells in the tumour microenvironment^28^. Thus, it is likely that multiple compartments contribute to IFN type-I pathway activation that we measured in TSA tumours after treatment with 8GyX3 (Fig. 1c). IFNβ production by phagocytes and endothelial cells could be triggered by tumour-derived DNA, or via Toll-like receptor stimulation by other signals released during immunogenic cell death induced by radiation^22^,^29^. However, our data unequivocally demonstrate that the abscopal effect is abrogated when cancer cells in the irradiated tumour do not express cGAS/STING or overexpress Trex1, which preclude cancer cell-intrinsic activation of IFN-I pathway (Figs 5 and 6 and Supplementary Fig. 5).

**Figure 9:**
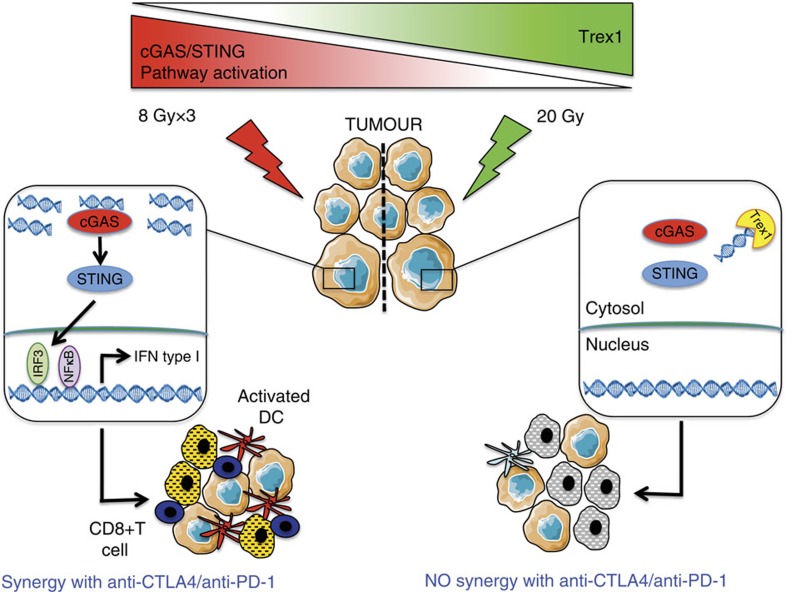
Graphical summary. Left, treatment with a radiation regimen that causes dsDNA accumulation in the cancer cells cytosol without inducing the DNAse Trex1 activates interferon type-I pathway via cGAS/STING. Downstream recruitment of DC and activation of CD8^+^ T cells is enabled and tumour rejection occurs in synergy with anti-CTLA4 or anti-PD-1 antibody. Right, in tumour treated with a dose of radiation above the threshold for Trex1 induction dsDNA is cleared from the cytosol precluding interferon-β release by the cancer cells. This leads to insufficient DC recruitment and activation and lack of CD8^+^ T cell activation resulting in absence of local and abscopal tumour regression in combination with immune checkpoint inhibitors.

We identified Trex1 as a key regulator of radiation-induced immunogenicity, and found that its expression is induced at levels sufficient to degrade cytosolic DNA by single doses of radiation with a threshold ranging from 12 to 18 Gy in different mouse and human carcinoma cells (Figs 7 and 8). As the radiation dose increases, more cytoplasmic dsDNA accumulates until the point is reached when Trex1 is markedly upregulated to degrade it. Thus, the balance between levels of dsDNA and Trex1 dictates IFN-stimulatory DNA accumulation in the cytoplasm of irradiated cells, and the subsequent development of anti-tumour T cell responses (Fig. 9). These data suggest a link between the immune-stimulatory effects of radiation and the DNA damage response, which is mediated via canonical pathways that regulate autoimmunity and the response to viral infections^17^,^30^,^31^.

Although a variety of radiation doses and delivery schedules have been used to induce anti-tumour T cells in different mouse tumour models^7^,^8^,^9^, abscopal responses reported in patients treated with radiation and anti-CTLA4 have been achieved with doses of <10 Gy repeated three to five times^3^,^4^,^5^,^32^. This suggests that radiation used at single doses that avoid Trex1 induction is more likely to synergize with immune checkpoint inhibitors in patients. The dose threshold for Trex1 induction in primary human tumours may differ. We found marked upregulation of *Ifnb1* and *Mx1* by 8GyX3, while 20 Gy upregulated *Trex1* in a patient-derived TP53/KRAS-mutated lung adenocarcinoma xenograft (Fig. 8c). Such PDTX-based testing could be used to identify the optimal radiation dose for individual patients, and personalize the radiation treatment in trials testing combinations of radiotherapy with immune checkpoint inhibitors and other immunotherapies, which are under investigation in large number of trials^33^.

Recently, loss of STING and/or cGAS expression has been reported in over a third of colorectal cancers^34^. STING was also shown to be frequently inactivated in HPV^+^ cancers^35^, and in a significant portion of primary and metastatic melanomas^36^. In some cases, other alterations downstream of STING precluded cancer cells from activating the transcription factors IRF3 or NF-κB^36^. Our data demonstrate that cGAS and STING expression, and function in the cancer cells are essential for optimal anti-tumour T cell induction by radiotherapy, suggesting their potential role as predictors of tumour response to combinations of radiation and immune checkpoint inhibitors. While the frequency of alterations in cGAS and STING in tumours other than colorectal and melanoma remains to be ascertained, transcriptional repression by promoter hypermethylation is usually observed^34^,^36^, suggesting the therapeutic potential for demethylating agents to rescue responsiveness to radiation and immune checkpoint inhibitors. Importantly, both the expression of cGAS and STING, and the functionality of the IFN-I pathway could be tested in PDTX, as we have shown here (Fig. 8c), providing the first biomarkers to tailor treatment and significantly advance the rapidly expanding field of radiation and immunotherapy combinations.

Finally, our data suggest a role for Trex1 as a therapeutic target. By controlling the amount of IFN-stimulatory DNA present in cancer cells, Trex1 is likely to reduce also IFNβ production by DCs activated by tumour-derived DNA, precluding spontaneous and radiation-induced T cell activation in immunogenic tumours infiltrated by sufficient numbers of DCs^21^,^22^. Thus, tumour-expressed Trex1 is an attractive target for interventions to improve in situ vaccination by radiotherapy and by other DNA damaging agents.

## Methods

### Mice

Wild type BALB/c and C57BL/6 mice were purchased from Taconic Animal Laboratory (Germantown, NY, USA). C57BL/6 *Ifnar1*^−/−^ mice were purchased from Mutant Mouse Research and Resource Center (MMRRC) at JAX and bred in house. BALB/c *Ifnar1*^−/−^ mice were a gift of Dr Joan Durbin, Rutgers, the State University of New Jersey. BALB/c *Batf3*^−/−^ mice were purchased from Jackson and bred in house. NOD/SCID/gamma (NOG) female mice (CIEA NOG mouse; NOD.Cg-*Prkdc*^*scid*^ *Il2rg*^*tm1Sug*^/JicTac) were purchased from Taconic Animal Laboratory (Germantown, NY, USA). All female and male mice were maintained under pathogen-free conditions in the animal facility at New York University School of medicine and Weill Cornell Medicine and used between 6 and 10 weeks of age. All experiments were approved by the Institutional Animal Care and Use Committee at both institutions.

### Cells and reagents

BALB/c mouse-derived poorly immunogenic mammary carcinoma 4T1 and TSA cells were obtained from F. Miller^37^ and P.L. Lollini^38^, respectively. C57BL/6 mouse-derived poorly immunogenic colorectal carcinoma MCA38 was a gift of Alan Frey^39^. MDA-MB-231 and 4175TR human triple negative breast cancer cells were purchased from ATCC and obtained from J. Massague, respectively. All cells were authenticated by IDEEX Bioresearch (Columbia, MO, USA), and further by morphology, phenotype, growth and pattern of metastasis *in vivo* and routinely screened for *Mycoplasma* (LookOut Mycoplasma PCR Detection kit, Sigma-Aldrich). None of the cells used are listed in the International Cell Line Authentication Committee (ICLAC) database of commonly misidentified cell lines. TSA, 4T1, MCA38 and MDA-MB-231 and 4175TR were cultured in DMEM (Life Technologies) supplemented with 2 mmol l^−1^ L-glutamine, 100 U ml^−1^ penicillin, 100 μg ml^−1^ streptomycin, 2.5 × 10^−5 ^mol l^−1^ 2-mercapthoethanol, and 10% FBS (Life technologies).

Anti-mouse CTLA4 monoclonal antibody (mAb) clone 9H10 (10 mg kg^−1^; Cat # BE0131), Syrian hamster IgG isotype control (10 mg kg^−1^; Cat # BE0087), anti-CD8 mAb clone 2.43 (5 mg kg^−1^; Cat # BE0061) and anti-PD-1 mAb clone RMP1-14 (10 mg kg^−1^; Cat # BE0146) were purchased from BioXCell. For experiments with doxycycline, cells were grown in media containing tetracycline-free fetal bovine serum and induced with 4 μg ml^−1^ of doxycycline 4 days prior to treatment. Recombinant human interleukin-2 (rIL2) was obtained from the Biological Resources Branch, Developmental Therapeutics Program, Division of Cancer Treatment and Diagnosis, National Cancer Institute.

### Cells with selected gene upregulation and downregulation

HEK 293-FT cells were used to produce viruses upon transfection of the packaging plasmids pPAX2 and pMD2 and a pTRIPZ vector containing a tetracycline-inducible promoter driving the expression of a TurboRFP fluorescent reporter (GE Dharmacon technology, provided by Dr Robert Schneider). ShRNAs (Fig. 2b) directed against IFNAR1 (mouse-shRNA: GAGTGACACCTTGCTTGTTTAT), cGAS (Mb21d1, E330016A19Rik) (mouse-shRNA: CAGGATTGAGCTACAAGAATAT; human-shRNA: AAGGAAGGAAATGGTTTCCAAG), STING (Tmem173, 2610307O08Rik, ERIS, MPYS, Mita) (mouse-shRNA: CTCGAAATAACTGCCGCCTCAT; human-shRNA: GGGCACCTGTGTCCTGGAGTAC) *Trex1* (mouse-shRNA TGCTCAGCATCTGTCAGTGGAG) or a non-silencing sequence (NS; mouse-human-shRNA: AATTCTCCGAACGTGTCACGT) were cloned into pTRIPZ using EcoRI and XhoI restriction sites. TSA, 4T1, MCA38 and MDA-MB-231 cells were transduced with cell-free virus-containing supernatants and selected with 4 μg ml^−1^ of puromycin during 48 h. Cells were further submitted to an RFP sorting to establish derivatives with stable expression of the shRNA constructs.

The pTRIPZ plasmid was modified to insert the mouse *Trex1* cDNA under the tetracycline-inducible promoter using AgeI and MluI restriction sites (Supplementary Fig. 3b) and used to transduce TSA cells (TSA^KI *Trex1*^).

### Tumour challenge and treatment

TSA cells (1 × 10^5^) and its derivatives (TSA^shIfnar1^, TSA^shcGAS^, TSA^shSTING^, TSA^shNS^ or TSA^KI *Trex1*^) were injected s.c. in the right flank of WT or *Ifnar1*^−/−^ or *Batf3*^−/−^ BALB/c mice on day 0 (primary tumour). On day 2, the contralateral flank was injected s.c. with parental TSA cells or TSA^shNS^ (abscopal tumour). MCA38 cells (5 × 10^5^) were injected s.c. in the right flank of WT or *Ifnar1*^−/−^ C57BL/6 mice on day 0. On day 2, the contralateral flank was injected s.c. with MCA38 cells (abscopal tumour). NOG mice were injected s.c. in the right flank on day 0 with 5 × 10^5^ MDA-MB-231 cells.

Perpendicular tumour diameters were measured with a vernier caliper, and tumour volumes were calculated as length × width^2^ × 0.52. When applicable, gene knockdown or knock-in expression were induced by adding doxycycline at 100 μg ml^−1^ (20 mg kg^−1^ day^−1^) into the mice drinking water at day 8, after tumours establishment. Doxycycline was replenished every 4 days until day 26. On day 12, when primary tumours reached an average size of 60–80 mm^3^, animals were randomly assigned to different treatment groups. Radiotherapy (RT) was delivered to the tumour as previously described^40^. Briefly, all mice (including mice receiving sham radiation) were anaesthetized by intra-peritoneal (i.p.) injection of avertin (240 mg kg^−1^) and the primary tumours irradiated with a single fraction of 8 Gy, 20 Gy or 30 Gy on day 12 or with 3 fractions of 8 Gy on day 12, 13 and 14 using the Small Animal Radiation Research Platform (SARRP Xstrahl, Surrey, UK). Anti-mouse CTLA4 mAb or its isotype control mAbs were administered i.p. (200 μg per mouse) on days 14, 17 and 20. Anti-mouse PD-1 mAb was given i.p. (200 μg per mouse) on days 12, 15, 19, 22 and 26. Anti-mouse CD8 mAb was administered i.p. (100 μg per mouse) on days 9, 10, 11, 17 and 23.

### Patient-derived tumour xenograft

Triaged seeds (0.1 × 0.3 × 0.3 cm) from established Patient-derived tumour xenograft (PDTX) from freshly resected primary lung tumour were implanted in a subcutaneous pocket on the flank areas of NOD.Cg-Prkdcscid B2mtm1Unc Il2rgtm1Wjl/SzJ mice (NSG) mice. Tumour growth was evaluated over time by visual inspection and tumour masses measured by an electronic caliper. Implanted PDTX are routinely excised (<2 cm^3^) and evaluated by histology, immunohistochemistry, target genomic sequencing and RNAseq. Written informed consents were collected in preoperative setting. PDTX studies were approved by Institutional Review Board of (IRB, 1410015560) of Weill Cornell Medicine.

### *In vivo* fluorescent imaging

Mice bearing RFP-tumours were anaesthetized with isoflurane to perform *in vivo* fluorescent images with IVIS Lumina III *in vivo* imaging system (Perkin Elmer). The photon radiance on the surface of an animal was expressed as photons per second per centimetre squared per steradian. Images shown are compound pictures generated by Living Images software (Supplementary Fig. 5b).

### Genome-wide microarray analysis

TSA tumours growing in WT BALB/c mice were excised 24 h after tumour-directed irradiation with a single dose of 20 Gy or three doses of 8 Gy given in consecutive days. Total RNA was purified with Qiagen RNeasy Mini kit. Quality and quantity of the Total RNA sample was assessed using an Agilent Bioanalyzer with the RNA6000 Nano Lab Chip (Agilent Technologies; Santa Clara, CA, USA). Labelled cDNA was prepared by linear amplification of the Poly(A)+ RNA population within the Total RNA sample. Briefly, Total RNA was reverse transcribed after priming with a DNA oligonucleotide containing the T7 RNA polymerase promoter 5′ to a d(T)24 sequence. After second-strand cDNA synthesis and purification of double-stranded cDNA, *in vitro* transcription was performed using T7 RNA polymerase. The quantity and quality of the cDNA was assayed by spectrophotometry and on the Agilent Bioanalyzer as indicated for Total RNA analysis. Purified cDNA was fragmented to uniform size and applied to Agilent Mouse Gene Expression 4 × 44 v2 Microarray (Agilent Technologies, design ID 026655) in hybridization buffer. Arrays were hybridized at 37 °C for 18 h in a rotating incubator, washed, and scanned on an Agilent G2505B Microarray Scanner. The data obtained have been deposited in the Gene Expression Omnibus (GEO) database (GSE83915).

All arrays were processed with Agilent Feature Extraction software and data was analysed with GeneSpring GX software (Agilent Technologies). To compare individual expression values across arrays, raw intensity data was quantile normalized across 18 samples and further normalized to the 0 Gy control for each set. Probes with intensity values above background in all samples within each group were used for further analysis. Differentially expressed probes were identified by >2-fold change and paired *t*-test *P* values<0.05 between each treatment group and its control. Data were further analysed using Gene Ontology classification and Ingenuity Pathway analysis. Both showed that immune response genes were the top categories of differentially expressed genes between 3 × 8 Gy and 20 Gy-treated tumours.

### Gene expression analysis

For analysis of tumour tissue total RNA was extracted after the homogenization of the samples using miRNeasy Mini Kit (QIAGEN, Germany, Cat # 217004) according to the manufacturer's instructions. Extracted RNA was subjected to complementary cDNA synthesis using RT2 preamp cDNA synthesis Kit (Cat # 330401) according to the manufacturer's instructions. Quantitative polymerase chain reaction with reverse transcription (qRT-PCR) was performed using RT2 SYBR Green qPCR Master Mix. The reaction (25 μl) along with cDNA was aliquoted into the wells of Custom array PCR array (Part # 4391528) which contains pre-dispensed, laboratory verified, specific primer pairs. Thermal cycling conditions (Applied Biosystems) includes the holding stage at 95 °C for 10 min followed by 40 cycles of each PCR step (denaturation) 95 °C for 15 s and (annealing/extension) 60 °C for 1 min. A melt curve analysis was also done to ensure the specificity of the corresponding RT-PCR reactions. For data analysis, the Ct values were exported to an Excel file and uploaded into the RT^2^ PCR Array data analysis web portal at https://www.qiagen.com/dataanalysiscenter in order to calculate fold change after normalization.

For analysis of TSA, MCA38, 4T1, MDA-MB-231, 4175TR cells and their derivatives total RNA was extracted from TRIzol lysates. For experiments testing the induction of Trex1 an initial kinetics analysis showed that Trex1 was upregulated in TSA cells very early following irradiation with 20 Gy reaching a plateau within 24 h, while no upregulation was seen with 8 Gy up to 72 h post radiation. Thus, 24 h was chosen as time point to test the effects of various radiation doses. Real-time PCR was performed using the Applied Biosystems 7500 real-time PCR cycler (ThermoFisher). One microgram of RNA was used for cDNA synthesis performed with the SuperScript VILO cDNA Synthesis Kit (ThermoFisher Scientific) followed by real-time RT-PCR with iTaq Universal SYBR Green Supermix (BioRad) according to manufacturer's protocol. Samples were normalized to housekeeping genes, and expression on untreated cells was assigned a relative value of 1.0. The PrimePCR SYBR Green assay primers (BioRad) used in this study were UniqueAssay ID: qMmuCED0050444 for mouse *Ifnb1*, qMmuCID0023356 for mouse *Mx1*, qMmuCED0046382 for mouse *Ifnar1*, qMmuCED0046127 for mouse *Sting*, qMmuCID0025813 for mouse *cGAS*, qMmuCED0001068 for mouse *Cxcl10*, qMmuCED0061616 for mouse *Trex1*, qMmuCED0041128 for mouse *Rpl13a*, qMmuCED0027497 for *Gapdh,* qHsaCID0010565 for human *Sting,* qHsaCID0009796 for human *cGAS*, qHsaCED0048594 for human *Trex1*, qHsaCED0045063 for human *Rpl13a and* qHsaCED0038674 for human *Gapdh*. Data were analysed with the 7500 Dx Instrument's Sequence Detection software (ThermoFisher). To calculate the relative gene expression, the 2(−ddCt) method was used.

### Measurement of IFN-β secretion

IFNβ was measured in cell-free supernatants collected 24 h after completion of tumour cells irradiation or lentiviral infection^41^ using the mouse (Cat # EPX01A-26044) or the human (Cat # EPX01A-12088) procarta kits (Affymetrix—eBioscience). Measured concentration was normalized by the number of viable cells.

### Flow cytometry analysis

Five days after the last radiation exposure, single cell suspensions obtained from collagenase-digested tumours were stained with the following antibodies purchased from Affymetrix—eBioscience: fixable viability dye efluor 780, CD40-FITC, CD70-APC, CD8α-PE efluor 610, CD45-Alexa fluor 700, CD11c-efluor 450. All samples were acquired with LSRII flow cytometer and analysed with FlowJo software (version 7.3.6).

### Analysis of IFN-γ production by CD8^+^ T cells

Overall, 5 × 10^5^ tumour-draining lymph node (TDLN) cells were stimulated *ex vivo* with 1 μM of the following peptides (GenScript): AH1A5 (SPSYVYHQF), or pMCMV (YPHFMPTNL). AH1A5 is a H2-L^d^-restricted T cell epitope derived from the gp70 Env product of an endogenous retrovirus, which is an immune-dominant target of CD8 T cells responses against TSA cells^38^. After 72 h of culture in 1 ml of T cell medium (RPMI 1640 medium supplemented with 2 mM L-glutamine, 100 U ml^−1^ penicillin, 100 μg ml^−1^ streptomycin, 50 μM 2-mercaptoethanil, 10% FBS) supplemented with 10 U ml^−1^ of human rIL2, cell-free supernatant were assessed for IFN-γ concentration using the procarta kit (Cat # EPX011-20606; Affymetrix—eBioscience).

### Western blot

Proteins from TSA, 4T1 or MDA-MB-231 parental cells and their derivatives were extracted in RIPA buffer (Sigma, Cat # R0278-50 ml) containing protease inhibitor cocktail (Sigma, Cat # 11836153001). Protein concentrations were determined with BCA method (Thermo Scientific, Cat # 23227). A total of 50 μg of protein of each sample was loaded in on 10% SDS-PAGE and run at 100 V constant voltage. Transfers of protein on an activated PVDF membrane (Millipore, Cat # IPVH07850) were performed overnight at 4 °C at 80 mA. PVDF membranes were probed with primary antibodies (m-anti-STING, Sigma, Cat # SAB1306152-40TST, 1:1,000; h-anti-STING, Sigma Cat # SAB1103582-200UL, 1:1,000; h-m-beta-actin, Cell Signaling Technology, Cat # 4967, 1:1,000) at 4 °C overnight. After washing three times, blots were then incubated with ECL anti-rabbit IgG secondary antibody (Sigma—Cat # GENA934-1 ml; 1:5,000) at room temperature for 30 min. Chemiluminescence was used to visualize protein bands (Perkin Elmer, Cat # NEL103001EA). Pictures were acquired using the Azure c600 imager (Azure Biosystems). Precision Plus Protein Dual Colour standard was used to estimate molecular weight (BioRad—Cat # 1610374). Bands of 25, 37 and 50 kDa were identified and marked on the film prior to scanning the gel. Unprocessed original scans of western blots are shown in Supplementary Fig. 7. Relative quantification was performed using ImageJ software to determine the STING/beta-actin ratio for each sample.

### Immunostaining of tumour sections

RFP+TSA tumours were fixed for 1 h at 4 °C in a 4% paraformaldehyde (PFA) in PBS, incubated overnight in 30% sucrose solution, and frozen in optimum cutting temperature (OCT) medium. Sections were incubated with 0.1% Tween 20 and 0.01% Triton X-100 for 20 min, followed by blocking with 4% rat serum in 4% bovin serum albumin (BSA)/PBS and staining with Alexa fluor 488 rat anti-mouse CD8α (ThermoFisher Scientific, Cat # MCD0820) for 90 min at RT. Mounting medium containing 4′,6-diamidino-2-phenylindole (DAPI) was used (Vector Laboratories, Cat # H-1500). Images were obtained using an EVOS FL microscope from Life Technologies. CD8 T cells were counted in five randomly selected (× 200) fields in each tumour.

### Immunostaining of cytoplasmic dsDNA

Doxycycline-induced TSA^shNS^ and TSA^shTrex1^ cells were exposed to 0 Gy, 8 Gy or 20 Gy. 24 h after radiation, cells were fixed for 10 min at room temperature in 4% PFA in PBS. Cells were permeabilized with 0.1% Tween 20 and 0.01% Triton X-100 for 20 min, followed by blocking with 1% BSA, 22.52 mg ml^−1^ glycine in PBST (0.1% Tween 20 in PBS) for 30 min at RT. Cells were incubated with a primary anti-dsDNA antibody (Abcam. Cat # ab27156; 1:1,000) overnight at 4 °C. After three washes, samples were incubated with a secondary antibody (goat anti-mouse IgG H&L—Alexa fluor 488 preabsorbed, Abcam, Cat # ab150117; 1:200) for 1 h at RT. After washes, mounting medium containing DAPI was used (Vector Laboratories, Cat # H-1500) to counterstain nuclei. Images were obtained using an EVOS FL microscope from Life Technologies.

### Quantification of cytoplasmic dsDNA

Cytoplasmic extracts from live cells determined by trypan blue exclusion were isolated using NE-PER Nuclear and Cytoplasmic Extraction Reagents (ThermoFisher Scientific, Cat # 78833). dsDNA was quantified in cytoplasmic fraction of 1 × 10^6^ live TSA cells 24 h after the last RT exposure using the SpectraMax Quant AccuClear Nano dsDNA Assay kit (Molecular devices, Part # R8357). The samples were read using the FlexStation 3 multi-mode microplate reader.

### Statistical analysis

On the basis of past experience and assuming a normal distribution and a coefficient of variation of approximately 25% for tumour growth rate, we determined that at least seven mice per group would be necessary to have 80% power at the two-sided 5% significance level to detect difference in the end-points evaluated^12^,^40^. Animals were randomly assigned to groups with no specific method of randomization and attempts were made to balance the groups so as to minimize the influence of other variables such as age or gender. No blinding was done during the *in vivo* studies. No animals were excluded from analysis.

Unless otherwise indicated, data are presented as mean±s.e. of the mean (s.e.m.). For comparisons with only two groups, *P* values were calculated using unpaired Student's two-tailed *t*-tests. Survival differences were assessed using log-rank tests. The two-way analysis of variance (ANOVA) test was used for tumour growth curve analyses. The Kaplan–Meier method was used to estimate median survival times. The s.e.m. are indicated by errors bars for each group of data. Differences were considered significant at *P* values below 0.05. All data were analysed using GraphPad Prism software (GraphPad version 6) with the exception of the genome-wide microarray data.

### Data availability

Microarray data were submitted to NCBI Gene Expression Omnibus (GEO) with accession number GSE83915. All other data generated or analysed during this study are included in this published article (and its Supplementary Information files) and raw data are available from the corresponding author upon reasonable request.

## Additional information

**How to cite this article:** Vanpouille-Box, C. *et al*. DNA exonuclease Trex1 regulates radiotherapy-induced tumour immunogenicity. *Nat. Commun.* **8**, 15618 doi: 10.1038/ncomms15618 (2017).

Publisher's note: Springer Nature remains neutral with regard to jurisdictional claims in published maps and institutional affiliations.

## Supplementary Material

Supplementary Information Supplementary Figures

Peer Review File
