# Supplementary material for: DNA exonuclease Trex1 regulates radiotherapy-induced tumour immunogenicity
Source: Nat Commun. 2017 Jun 9;8:15618. doi: 10.1038/ncomms15618 (PMC5472757; doi:10.1038/ncomms15618)
Supplement: Supplementary Information — Supplementary Figures [file ncomms15618-s1.pdf]

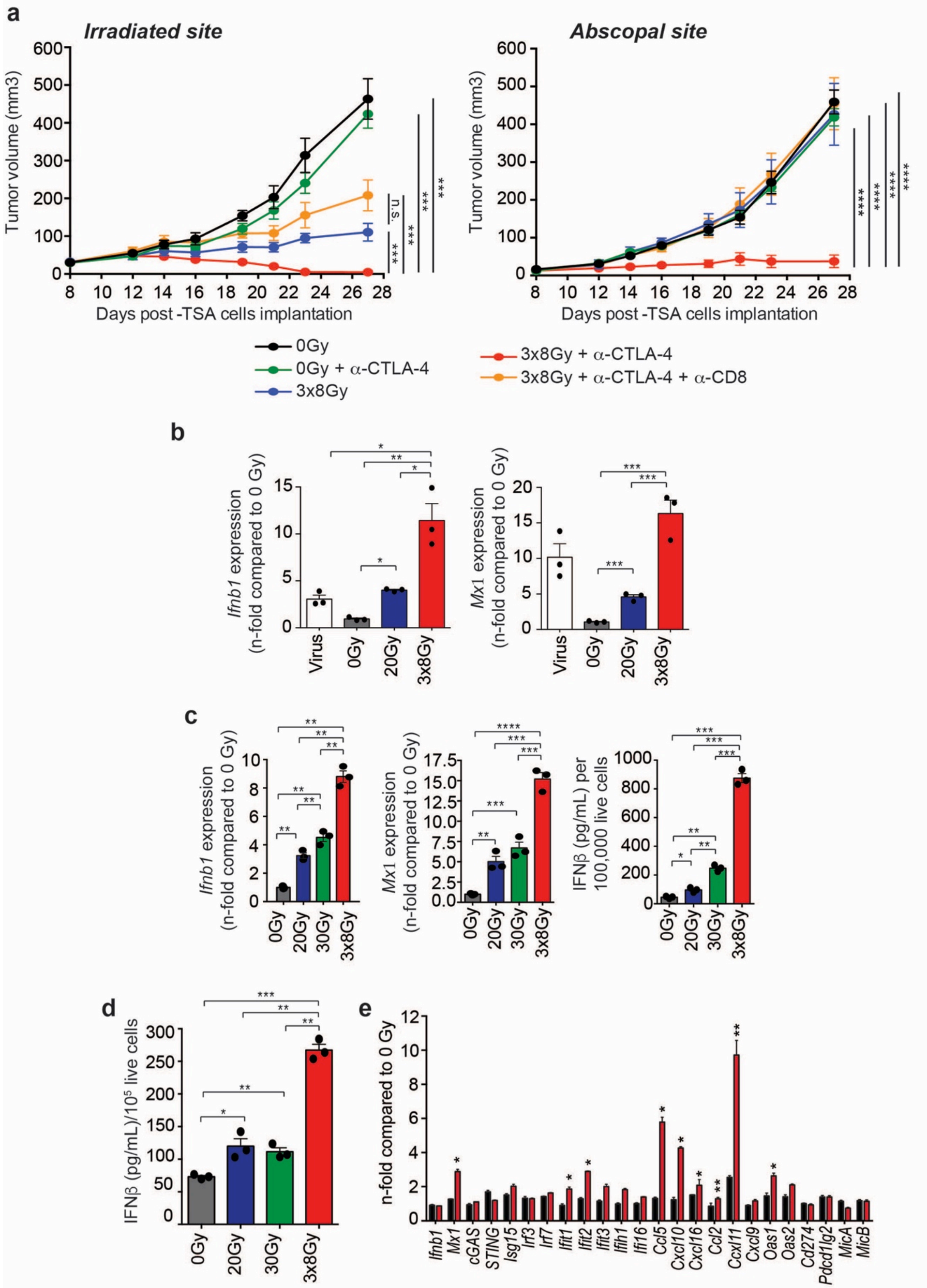

Supplementary Figure 1: Cancer cell-intrinsic radiation-induced activation of type-I interferon pathway is seen in multiple cancer cell and tumors models. (**a**) The therapeutic effect of 8GyX3+anti-CTLA4 is CD8-dependent. Growth of irradiated and abscopal TSA tumors in BALB/c mice treated with 0Gy (black solid line), 0Gy+anti-CTLA-4 (green line), 8GyX3 (blue line), 8GyX3 + anti-CTLA-4 (red line) and 8GyX3 + anti-CTLA-4 + anti-CD8 (yellow line), ****p*<0.0005;

***p<0.0001: comparison of tumor growth; two-way ANOVA; n=6. All data are mean ± s.e.m. (**b**) Mouse 4T1 breast carcinoma cells exposed to viruses or radiation were tested by qRT-PCR for *Ifnb1* and *Mx1* gene expression 24 hrs later (n=3/group). **(c)**, Mouse MCA38 colorectal carcinoma were tested by qRT-PCR for *Ifnb1* and *Mx1* gene expression and by ELISA for IFNβ secretion 24 hrs after radiation (Duplicate; *p<0.05; **p<0.005; ***p<0.0005: t-test; n=3). (**d**) Human MDA-MB-231 breast cancer cells exposed *in vitro* to radiation were tested for IFNβ secretion 24 hrs later (Duplicate; *p<0.05; **p<0.005; ***p<0.0005: t-test; n=3) (**e**). MDA-MB-231 tumors grown in NOG mice were tested by qRT-PCR for ISGs expression 24 hrs following *in vivo* radiotherapy with 20Gy (black bars) or 8GyX3 (red bars). (Duplicate; *p<0.05; **p<0.005; ***p<0.0005: t-test; n=4). All data are mean ± s.e.m.

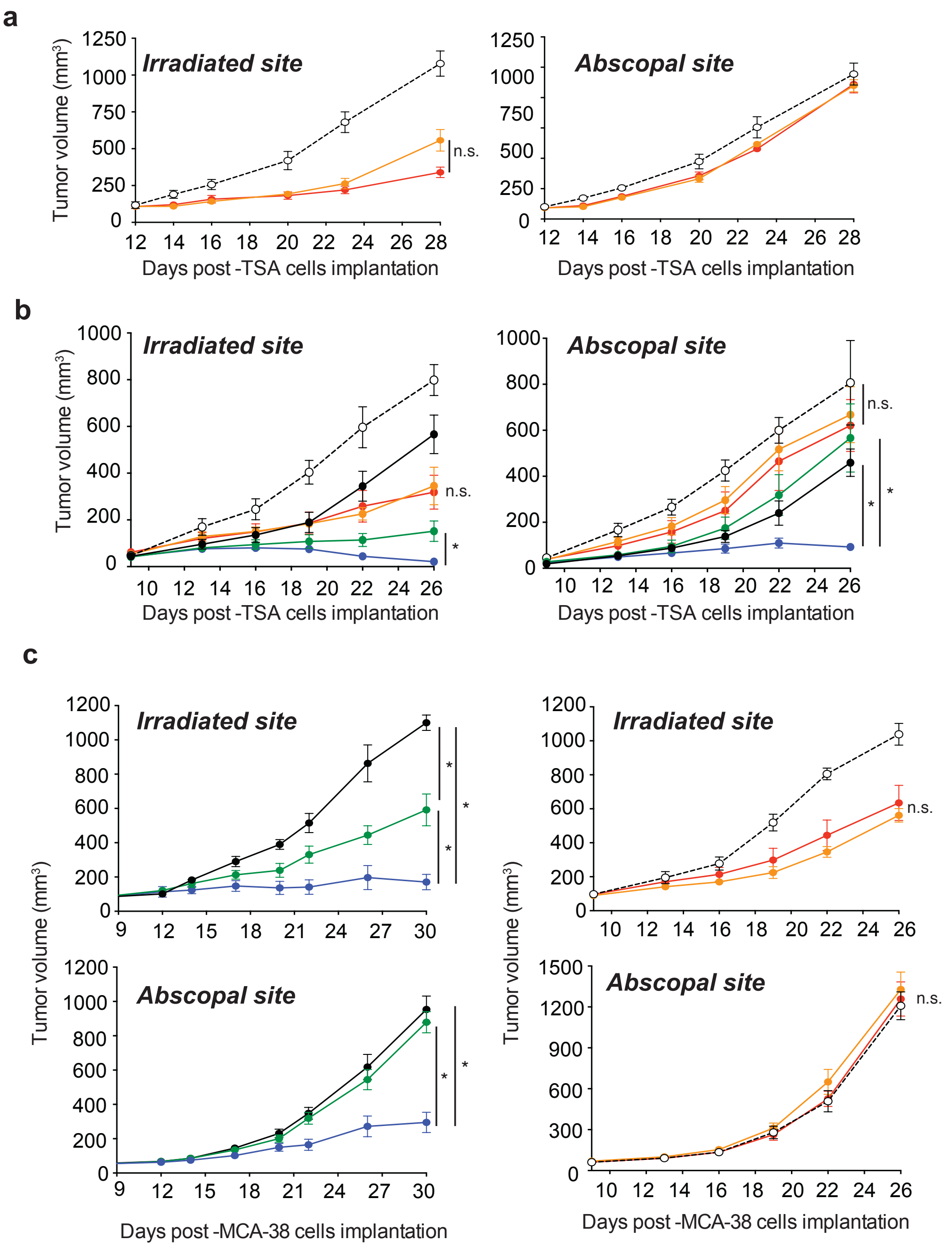

Supplementary Figure 2: Loss of therapeutic effect of 8GyX3 radiation and anti-CTLA4 in *Batf3*$^{-/-}$ and *Ifnar1*$^{-/-}$ mice. (**a**) Growth of irradiated and abscopal TSA tumors in BALB/c *Batf3*$^{-/-}$ mice treated with 0Gy (dashed line), 8GyX3 (yellow line), 8GyX3+anti-CTLA4 (red line). (**b**) Growth of irradiated and abscopal TSA tumors in wild-type BALB/c mice treated with 0Gy (black solid line), 8GyX3 (green line), 8GyX3+anti-CTLA4 (blue line) and in BALB/c *Ifnar1*$^{-/-}$ mice treated with 0Gy (dashed line), 8GyX3 (yellow line), 8GyX3+anti-CTLA4 (red line). (**c**) Growth of irradiated and abscopal MCA38 tumors in wild-type C57BL/6 treated with 0Gy (black solid line), 8GyX3 (green line), 8GyX3+anti-CTLA4 (blue line) and C57BL/6 *Ifnar*$^{-/-}$ mice treated with 0Gy (dashed line), 8GyX3 (yellow line), 8GyX3+anti-CTLA4 (red line). Duplicate; *$p<0.05$; **$p<0.005$: comparison of irradiated tumor outgrowth; two-way ANOVA; n=7. All data are mean ± s.e.m.
tumors (blue) remained tumor-free. All data are mean ± s.e.m.

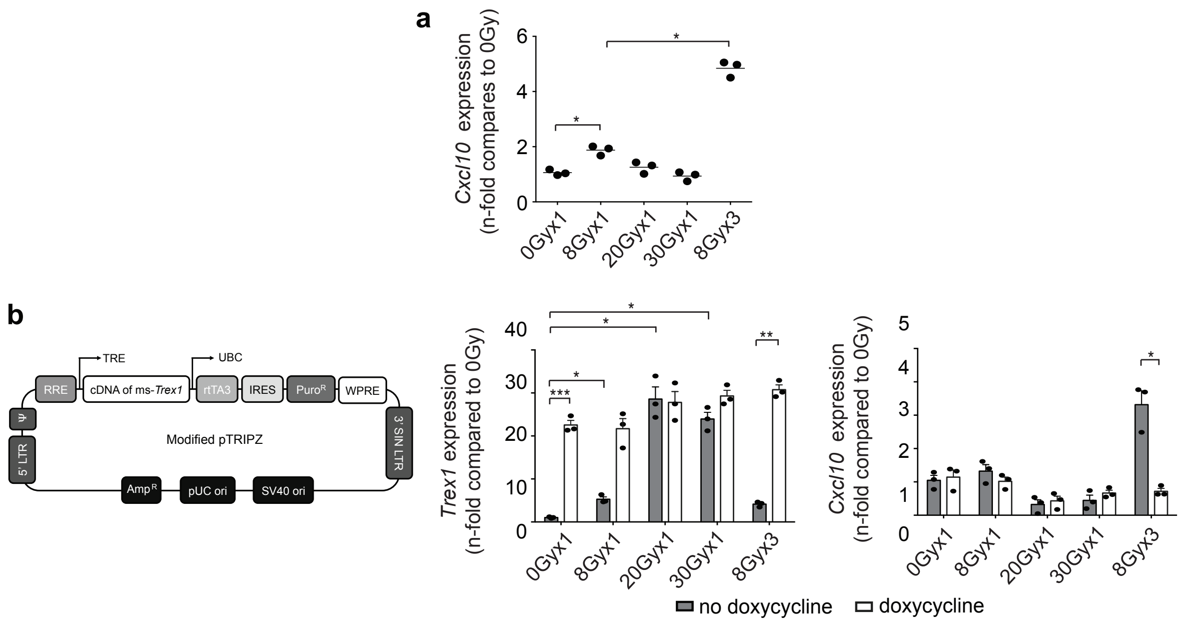

Supplementary Figure 3: **Forced expression of Trex1 abrogates cxcl10 induction by 8GyX3.** (**a**) *Cxcl10* gene expression in TSA cells treated with different radiation doses. Duplicate; **p*<0.05; ***p*<0.005; ***p<0.0005: *t*-test; n=3. (**b**) Modified pTRIPZ lentiviral vector used to transduce TSA cells with *Trex1* under the control of a tetracycline-inducible promoter ($TSA^{KI\ Trex1}$). *Trex1* expression is induced by doxycycline (white bars) at levels comparable to *Trex1* expression induced by high dose radiation (gray bars), as measured by qRT-PCR 24hr after radiation completion. Duplicate; **p*<0.05; ***p*<0.005; ***p<0.0005: *t*-test; n=3. Doxycycline-induced *Trex1* (white bars) abrogates the upregulation of *Cxcl10* by 8GyX3 measured (gray bars) in $TSA^{KI\ Trex1}$ cells, measured by qRT-PCR. Duplicate; **p*<0.05; ***p*<0.005; ***p<0.0005: *t*-test; n=3. All data are mean ± s.e.m.

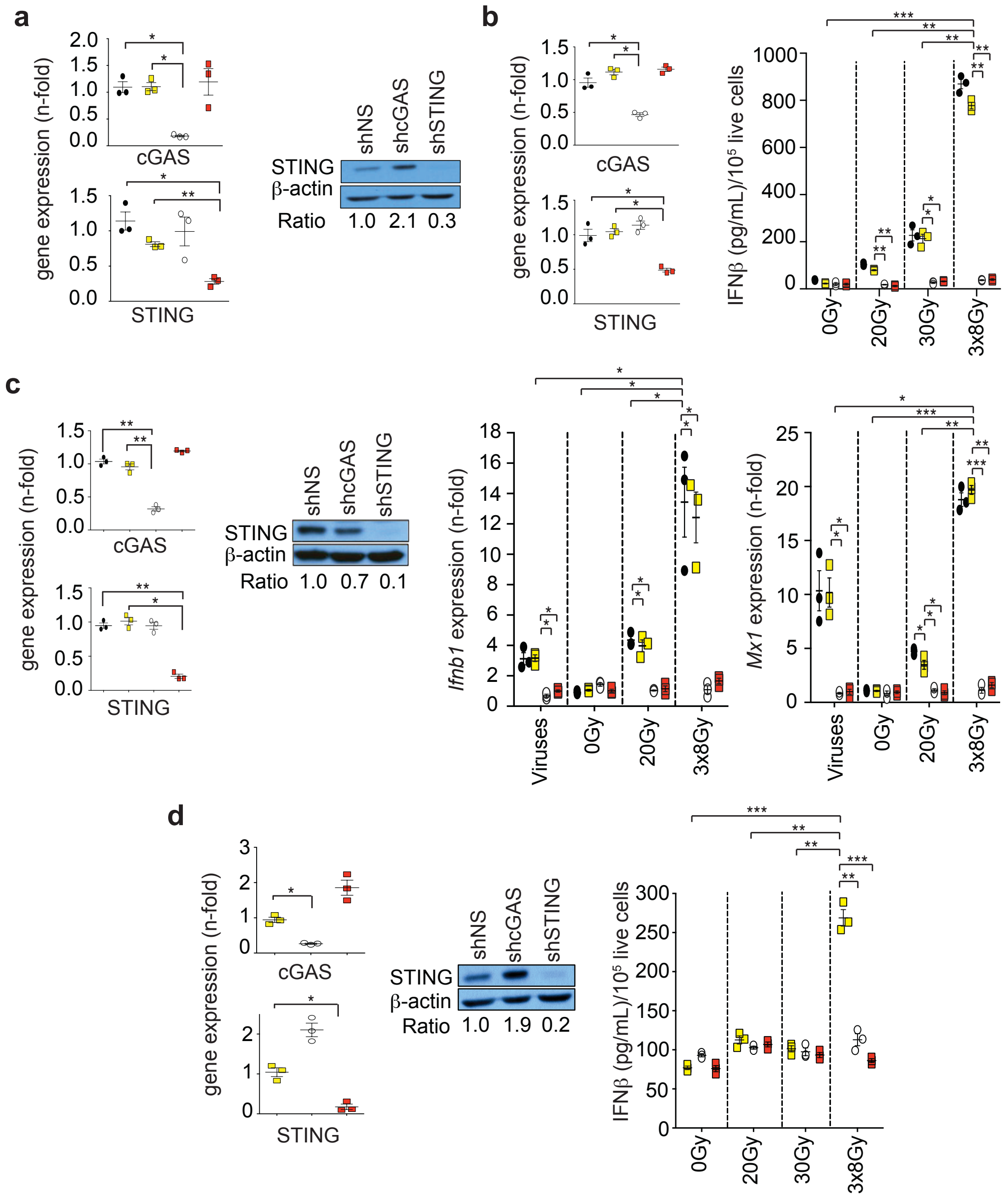

Supplementary Figure 4: Radiation-induced cancer cell intrinsic type I IFN activation is mediated by cGAS-STING in multiple tumors. (a-d) Parental cells (black circles), and cells transduced with non-silencing construct (yellow squares), shcGAS (white circles) and shSTING (red squares) cultured with doxycycline. (**a**) Selective and efficient knockdown of cGAS or STING in TSA cells by qRT-PCR and western blot. (**b**) Selective and efficient knockdown of cGAS or STING in MCA38 cells results in abrogation of radiation-induced IFNβ secretion. (**c**) Selective and efficient knockdown of cGAS or STING measured by qRT-PCR and western blot in 4T1 cells abrogates virus- and radiation-induced *Ifnb1* and *Mx1* expression. (**d**) Selective and efficient knockdown of cGAS or STING as measured by qRT-PCR and western blot in MDA-MB-231 cells. IFNβ secretion induced by 8GyX3 is abrogated in MDA-MB-231^{shcGAS} (white circles) and in MDA-MB-$231^{shSTING}$ (red squares). (**a-d**) Duplicate; *$p<0.05$; **$p<0.005$; ***$p<0.0005$: *t*-test; n=3. All data are mean ± s.e.m.

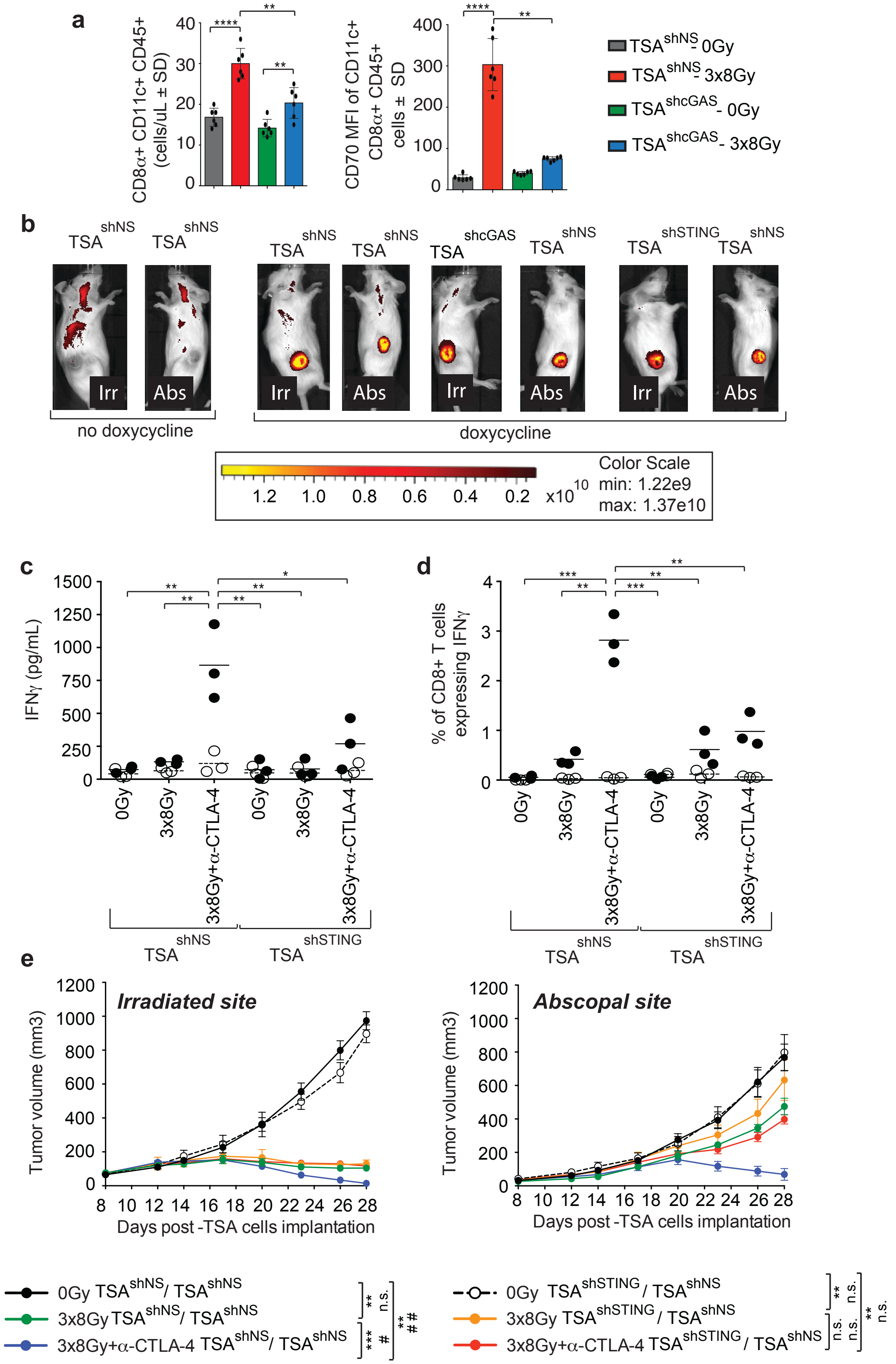

Supplementary Figure 5: *cGAS and STING* in TSA cancer cells are required for dendritic cell recruitment and radiation-induced anti-tumor immunity. (**a**) Absolute cell numbers of CD8α$^+$ CD11c$^+$ CD45$^+$ dendritic cells and their CD70 expression is increased by 8GyX3 in TSA^{shNS} but not TSA^{shcGAS} tumors 5 days after irradiation (Duplicate; **p*<0.05; ***p*<0.005; ***p<0.0005: *t*-test; n=6).

(**b**) In vivo induction of tRFP detected by fluorescence imaging of mice bearing TSA^{shNS}, TSA^{shcGAS}, or TSA^{shSTING}, in the irradiated tumor (Irr) and TSA^{shNS} in the abscopal tumor (Abs) upon doxycycline treatment. **(c-e)** Mice with TSA^{shSTING}, or non-silencing shRNA (TSA^{shNS}) in the irradiated tumor and TSA^{shNS} in the abscopal tumor were treated with doxycycline, 8GyX3 and anti-CTLA4. IFNγ production by TDLN (**c**) and percentage of IFNγ^{+} CD8^{+} T cells in spleen (**d**) in response to CD8 epitope AH1A5 (full circles) or control peptide MCMV (open circles). Each symbol represents one animal. Horizontal lines indicate the mean of antigen-specific (solid lines) or control (dashed lines). Growth of irradiated and abscopal tumor (**e**) in mice with TSA^{shNS} cells treated with 0Gy (black), 8GyX3 (green), 8GyX3+anti-CTLA4 (blue), and mice with TSA^{shSTING} cells treated with 0Gy (dashed line), 8GyX3 (yellow), 8GyX3+anti-CTLA4 (red). (**c-d**) Duplicate; *p<0.05; **p<0.005; ***p<0.0005: *t*-test; n=3. (**e**) Duplicate; *p<0.05; **p<0.005: comparison of irradiated tumor outgrowth; two-way ANOVA; n=7; $^{##}p$<0.005: comparison of abscopal tumor outgrowth; two-way ANOVA; n=7. All data are mean ± s.e.m.

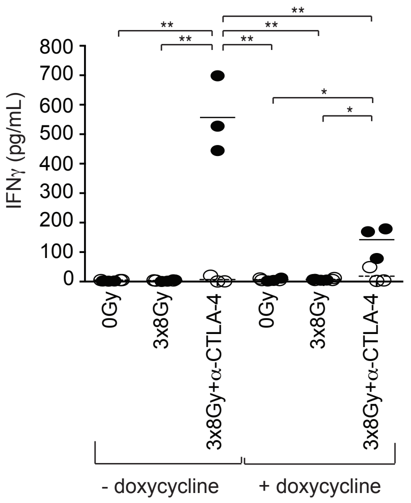

Supplementary Figure 6: Trex1 regulates radiation-induced priming of tumor-specific CD8+ T cells. Half of the mice with doxycycline-inducible *Trex1* in TSA cells (TSA$^{KI\ Trex1}$) in the irradiated tumor and parental TSA (TSA-WT) in the abscopal tumor were given doxycycline and all mice were then treated with 8GyX3 and anti-CTLA4. IFNγ production by TDLN cells in response to CD8 epitope AH1A5 (full circles) or control peptide MCMV (open circles). Each symbol represents one animal. Horizontal lines indicate the mean of antigen-specific (solid lines) or control (dashed lines) IFNγ concentration (Duplicate; *p<0.05; **p<0.005: *t*-test; n=3.).

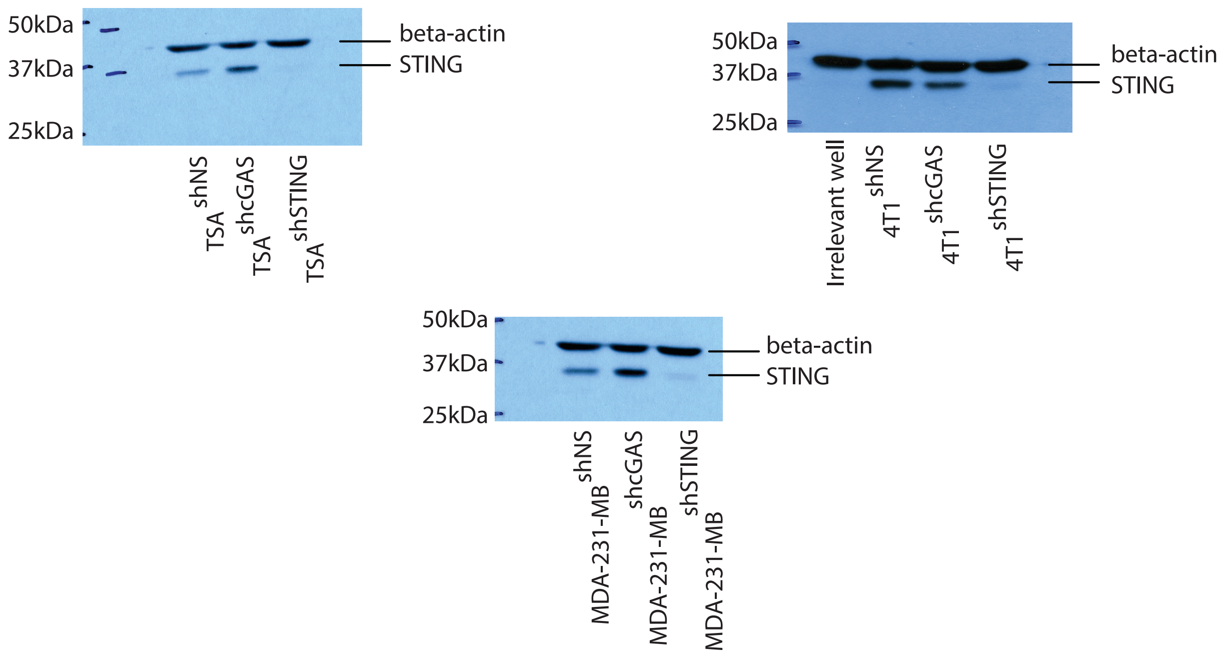

Supplementary Figure 7. Unprocessed original scans of western blots shown in Supplementary Figure 4.
