# Supplementary material for: DNA exonuclease Trex1 regulates radiotherapy-induced tumour immunogenicity
Source: Nat Commun. 2017 Jun 9;8:15618. doi: 10.1038/ncomms15618 (PMC5472757; doi:10.1038/ncomms15618)
Supplement: Peer Review File [file ncomms15618-s2.pdf]

Editorial Note: this manuscript has been previously reviewed at another journal that is not operating a transparent peer review scheme. This document only contains reviewer comments and rebuttal letters for versions considered at Nature Communications.
